# β-Sitosterol Attenuates Dexamethasone-Induced Muscle Atrophy via Regulating FoxO1-Dependent Signaling in C2C12 Cell and Mice Model

**DOI:** 10.3390/nu14142894

**Published:** 2022-07-14

**Authors:** Young-Sool Hah, Won Keong Lee, Sangyeob Lee, Eun Ji Kim, Jung Hyeon Lee, Seung-Jun Lee, Yeong Ho Ji, Sang Gon Kim, Hyeong-Hwan Lee, Seo Yeon Hong, Jun-Il Yoo

**Affiliations:** 1 Department of Orthopedics, Institute of Health Sciences, Gyeongsang National University School of Medicine, Jinju 52727, Korea; imhappyto@hanmail.net (Y.-S.H.); uranus-jh@hanmail.net (J.H.L.); 2 Biomedical Research Institute, Gyeongsang National University Hospital, Jinju 52727, Korea; lwk928@naver.com (W.K.L.); sylee2@gnu.ac.kr (S.L.); monsterun@naver.com (E.J.K.); 3 Department of Theriogenology and Biotechnology, College of Veterinary Medicine, Gyeongsang National University, Jinju 52828, Korea; 4 Department of Convergence of Medical Sciences, Gyeongsang National University, Jinju 52828, Korea; 0789zxc@gmail.com (S.-J.L.); wldudgh503@naver.com (Y.H.J.); 5 Anti-Aging Research Group, Gyeongnam Oriental Anti-Aging Institute, Sancheong 52215, Korea; sen600@hanmail.net (S.G.K.); furim0714@daum.net (H.-H.L.); 6 Division of Applied Life Science (BK21 PLUS), IALS, Gyeongsang National University, Jinju 52727, Korea; 7 Crop Production Technology Research Division, NICS, RDA, Miryang 50424, Korea; agriculture63@korea.kr

**Keywords:** β-sitosterol, FoxO1, dexamethasone, muscle atrophy, MuRF1, MAFbx

## Abstract

Sarcopenia refers to a decline in muscle mass and strength with age, causing significant impairment in the ability to carry out normal daily functions and increased risk of falls and fractures, eventually leading to loss of independence. Maintaining protein homeostasis is an important factor in preventing muscle loss, and the decrease in muscle mass is caused by an imbalance between anabolism and catabolism of muscle proteins. Although β-sitosterol has various effects such as anti-inflammatory, protective effect against nonalcoholic fatty liver disease (NAFLD), antioxidant, and antidiabetic activity, the mechanism of β-sitosterol effect on the catabolic pathway was not well known. β-sitosterol was assessed in vitro and in vivo using a dexamethasone-induced muscle atrophy mice model and C2C12 myoblasts. β-sitosterol protected mice from dexamethasone-induced muscle mass loss. The thickness of gastrocnemius muscle myofibers was increased in dexamethasone with the β-sitosterol treatment group (DS). Grip strength and creatine kinase (CK) activity were also recovered when β-sitosterol was treated. The muscle loss inhibitory efficacy of β-sitosterol in dexamethasone-induced muscle atrophy in C2C12 myotube was also verified in C2C12 myoblast. β-sitosterol also recovered the width of myotubes. The protein expression of muscle atrophy F-box (MAFbx) was increased in dexamethasone-treated animal models and C2C12 myoblast, but it was reduced when β-sitosterol was treated. MuRF1 also showed similar results to MAFbx in the mRNA level of C2C12 myotubes. In addition, in the gastrocnemius and tibialis anterior muscles of mouse models, Forkhead Box O1 (FoxO1) protein was increased in the dexamethasone-treated group (Dexa) compared with the control group and reduced in the DS group. Therefore, β-sitosterol would be a potential treatment agent for aging sarcopenia.

## 1. Introduction

Sarcopenia refers to a decline in muscle mass and strength with age, causing significant impairment in the ability to carry out normal daily functions and increased risk of falls and fractures, eventually leading to loss of independence [1,2]. Sarcopenia is not a simple loss of muscle mass and strength but represents a precursor of frailty and a predictor of increased mortality in chronic diseases [3,4,5,6,7,8]. Moreover, sarcopenia raises the risk of physical disability, poor life quality, and mortality, among other things [9]. So far, several studies have reported that the development of sarcopenia is related to multifactorial and interrelated physical inactivity, aging, nutritional factors, and biological markers and mechanisms such as sex hormones, oxidative products, and inflammatory pathways [10,11,12,13,14]. Of those mechanisms, chronic activation of the inflammatory response is the key physio-pathological substrate for anabolic resistance, sarcopenia, and frailty in older individuals [15].

β-sitosterol exists in various plant parts such as fruits, leaves, rhizomes, and a variety of plant tissue cultures [16]. It is one of the phytosterols with a similar chemical structure to cholesterol [17]. It has been confirmed that β-sitosterol administration up to 1000 mg/kg in rats and mice causes no cytotoxicity or genotoxicity [18]. Several studies showed that β-sitosterol has an antioxidant property by antioxidant enzymes and human estrogen receptors [19,20]. According to a study, β-sitosterol lowered oxygen free radical and hydrogen peroxide levels in RAW 264.7 cells treated with phorbol myristate acetate (PMA) [20]. In addition, β-sitosterol has been studied to be effective in various inflammation-related models [21]. There are studies showing that it is effective in colitis, intestinal inflammation, and obesity-related inflammation [22,23,24]. These results showed that β-sitosterol could be a potential agent in various diseases.

Muscle atrophy can be caused by aging, starvation, diabetes, or catabolic stimulation [25]. Among them, catabolic stimulation is an important factor in relation to muscle mass. This is because catabolism is related to the maintenance of protein, and muscle occupies a large proportion of the total body mass. Therefore, maintaining protein homeostasis is an important factor in preventing muscle loss [25]. The decrease in muscle mass is caused by an imbalance between anabolism and catabolism of muscle proteins [26]. The catabolism and anabolism of muscle protein can be attributed to factors associated with aging, such as lack of nutrition, physical inactivity, and hormonal changes [27]. In particular, the intracellular insulin signaling pathway and the insulin-like growth factor-1 (IGF-1) receptor pathway (IGF1/AKT/mTOR) are the primary regulators of anabolism [28]. Muscle mass loss via degradation of these muscle proteins is known to pass through the ubiquitin-proteasome pathway [29,30]. This condition includes a mechanism of muscle atrophy F-box (MAFbx) or a muscle ring-finger protein 1 (MuRF1) in recent studies [31,32,33]. Additionally, IGF-1 inhibits the expression of MAFbx and MuRF1, which are associated with the transcription factors, the FOXO family [34,35]. Until now, many factors have been introduced that show prevention effects on sarcopenia, such as vitamin D [36,37], antioxidant nutrients [38], anti-inflammatory agents [39,40], etc., but few studies have been conducted on muscle proteins catabolism or anabolism of these factors. Therefore, analyzing useful components through the above mechanism would be effective in preventing sarcopenia.

Recent research suggests that there is also an increase in catabolic signals with age, which causes muscle mass loss [41]. Although β-sitosterol has various effects such as anti-inflammatory, protective effects against NAFLD, antioxidant, and antidiabetic activity, few studies have applied it to aging-related muscle loss [42,43,44,45]. In addition, the mechanism of the β-sitosterol effect on the catabolic pathway was not well known. Therefore, we applied β-sitosterol to dexamethasone-induced muscle atrophy cells and animal models to investigate the role of sitosterol in the catabolism pathway caused by aging-related muscle loss.

## 2. Materials and Methods

### 2.1. Animal Study Design

The Animal Experimental Ethics Committee of Gyeongsang National University (GNU-180823-M0044) gave its approval to perform animal tests, and the research was carried out following the ethical protocol for animal experimentation. We purchased 6-week-old male C57BL/6 mice with an average body weight of 22 g from Core Tech Co., Ltd, (Seoul, Korea) for the experimental animals. These animals were used in the experiment after a week of adaptation in a light and dark cycle environment with a temperature of 24 ± 2 °C, relative humidity of 40–60%, an illuminance of 150–300 lux, and a 12-hour interval. Normal food was provided during the adaption period, and sterile water was freely provided with drinking water. After the adaptation period, C57BL/6 mice were randomly divided into four groups (*n* = 9 per group): control group (Control), β-sitosterol treatment group (S), dexamethasone treatment group (Dexa), and dexamethasone + β-sitosterol treatment group (DS).

After the experimental animal adaptation phase, dexamethasone (20 mg/kg of body weight of mice) was injected intraperitoneally into the Dexa and DS groups at 10~11 a.m. every day for two weeks to generate a muscle atrophy model. The saline injection was administered to the control group. The control and Dexa groups received no medications during the same period. From one week before dexamethasone treatment to the end of the experiment, the DS group was given β-sitosterol (S0040; Toyko Chemical Industry, Tokyo, Japan) 200 mg/kg body weight of mice orally once a day. Three days before and after dexamethasone treatment, body weights were measured. The total experimental period for treatment was 3 weeks. On the day of the end of the experiment, all groups were euthanized, and the tibialis anterior and gastrocnemius muscles were quickly frozen in liquid nitrogen and stored at −80 °C for use in identifying the target protein through western blot.

### 2.2. Grip Strength Test

The grip strength of all test animals was assessed the day before the end of the experiment. The Bioseb Grip Strength Test was used to determine grip strength in grams (BIO-GS3; BIOScience and Experimental Biology, Pinellas Park, FL, USA). The grip strength was assessed by tugging the tail at a steady speed (2 cm/s) until the grasp was released, and the stainless-steel T-bar of the experimental tool was gripped with both front paws of the test animal. The average value was calculated after each animal received five measurements.

### 2.3. Treadmill Analysis

Treadmill equipment (Panlab, Barcelona, Spain) was used to measure the speed, duration, and distance of the mouse running test, which was then changed using software (SeDaCom v2.0.02, Panlab, Barcelona, Spain). The adaptive gait speed was set to 10 cm/s for 3 min, then increased to 4 cm/s every 4 min until the maximum speed of 75 cm/s was attained, at which point the test was stopped. All groups were given the same adaptive walking and running speeds, as well as electric stimulation (1.1 mA) behind each treadmill rail to force them to run. The time to fatigue was calculated by placing the front legs on the rails for 3 s and the back legs on the electrical device for 3 s.

### 2.4. Histological Analysis of Muscle Tissue

The right tibialis anterior, gastrocnemius muscles, and extensor digitorum longus muscles were cryosectioned after being instantly frozen with Optimal Cutting Temperature (OCT) compounds (Lab-Tek; Miles Laboratories, Inc., Naperville, IL, USA). A cryostat (Leica CM1950; Heidelberg, Germany) was used to cut 5 mm thick muscle slices from the frozen samples, and 10 percent goat serum was used to block them for 1 h at room temperature. Wheat germ agglutinin, Alexa Fluor488 conjugate (W11261; Invitrogen/Thermo Fisher Scientific, Waltham, MA, USA) antibody was diluted to a concentration of 1:500 and stained at 4 °C overnight, and an upright microscope (Nikon Eclipse ni DSRi2; Nikon, Tokyo, Japan) was used to observe the extracellular matrix. The fiber cross-sectional area (CSA) and Min Feret diameter were measured with an ImageJ application after the samples were imaged with a microscope at 100 magnification.

### 2.5. In Vitro Study Design

Mouse C2C12 myoblast was purchased from the American Type Culture Collection (Manassa, VA, USA). C2C12 myoblasts were seeded at 3 × 10^5^/well on a 6-well culture plate containing 90% Dulbecco’s modified Eagle’s medium (DMEM), 10% fetal bovine serum (FBS), 100 units/mL penicillin and streptomycin (PS), and cultured at 37 °C CO_2_. Myoblasts were differentiated to myotube cells using a differentiation medium containing 2 percent horse serum (HS) and 100 unit/mL PS, which was cultured for 7 days with the differentiation medium being changed every 2 days. As a muscle reduction cell model, muscular atrophy was generated by treating cells with dexamethasone (1 μM) for 48 h. At this time, β-sitosterol was mixed for 48 h on the 5th day. All in vitro data were obtained from multiple experiments.

### 2.6. Cell Viability

C2C12 cells were seeded in 24-well plates at a concentration of 5 × 10^4^ cells per well. After 24 h incubation, the specified dose of β-sitosterol or dimethyl sulfoxide (DMSO) was added to the medium for 24 h. Afterwards, the cells in each well were treated according to the instructions of the Cell Counting Kit-8 (Dojindo Laboratories, Kumamoto, Japan). Briefly, 10 μL per well of the CCK-8 solution was added and incubated for 1 h at 37 °C in a humidified, 5% CO_2_ atmosphere. The amount of formazan dye generated by cellular dehydrogenase activity was measured by absorbance at 450 nm with a microplate reader (Molecular Devices, San Jose, CA, USA).

### 2.7. Western Blot Analysis

Cells and tissues were washed twice with cold phosphate buffered saline (PBS) buffer before being lysed in radioimmunoprecipitation assay (RIPA) lysis buffer (10 mM Tris-Cl, pH 7.4, 150 mM NaCl, 1 mM EDTA, 1% Triton X-100, 1% sodium deoxycholate, 0.1 percent SDS) supplemented with phenylmethylsulfonyl fluoride (PMSF), protease inhibitor cocktail, and sodium orthovanadate (Santa Cruz Biotechnology, Santa Cruz, CA, USA) for 10 min. Homogenization was performed once again using an ultrasonic grinder. The homogenate was centrifuged at 13,000× *g* for 15 min at 4 °C before being measured using the BCA Protein Assay Kit (Pierce, Rockford, IL, USA). The protein was measured and transferred to a nitrocellulose membrane after electrophoresis on a 10% SDS polyacrylamide gel at the same concentration (30 μg total protein). Each blot was incubated overnight with primary antibodies against MuRF-1 (sc-398608; Santa Cruz Biotechnology, Dallas, TX, USA), MAFbx (sc-166806; Santa Cruz Biotechnology, Dallas, TX, USA), FoxO1 (2880s; Cell Signaling Technology, Danvers, MA, USA), FoxO3 (2497s; Cell Signaling Technology), Gapdh (ATGA0394; ATGen, Montevideo, Uruguay), and β-actin (A5441; Sigma-Aldrich, St. Louis, MO, USA) after blocking with Tris-buffered saline containing 5% skim milk (Difco, Detroit, MI, USA) and 0.05 percent Tween 20 (TBST) at room temperature for 1 h. The membrane was rinsed three times with TBST the next day, then reacted for one hour with a peroxidase-conjugated secondary antibody before being washed three times with TBST. Each band was detected using Clarity Western ECL Substrate (Bio-Rad Laboratories, Inc., Berkeley, CA, USA) with the ChemiDocTM Touch Imaging System (Bio-Rad Laboratories, Inc., Hercules, CA, USA).

### 2.8. Total RNA Isolation and qPCR

Total RNA was extracted using Trizol solution after C2C12 myoblasts were washed twice with cold PBS. The iScriptTM cDNA Synthesis Kit (Bio-Rad, Hercules, CA, USA) was used to convert the extracted 2 μg RNA into cDNA, and mRNA expression was measured using the ViiATM7 Real-Time PCR System (Applied Biosystems, Waltham, MA, USA) and TaqMan analysis. Amplification was done under the following conditions: 95 °C, 10 min; 40 cycles at 95 °C, 15 s and 60 °C, 60 s. MuRF1 (Mm01185221 m1), MAFbx (Mm00499523 m1), MyoD (Mm00440387 m1), MyoG (Mm00446194 m1), and Myostatin (Mm01254559 m1) were used as primers in this study. After amplification, the data were evaluated 40 times at intervals.

### 2.9. Statistical Analysis

For statistical analysis, GraphPad Prism (Version 5.01; GraphPad Software, San Diego, CA, USA) was used. All the tests were carried out three times, and the results were expressed as mean ± SD or mean ± SE. To determine the significance between two groups, a Student’s *t*-test was used and among three or more groups, a one-way ANOVA analysis was used. A *p*-value of less than 0.05 was considered significant.

## 3. Results

### 3.1. β-Sitosterol Protects Mice from Dexamethasone-Induced Muscle Atrophy

**Figure 1:**
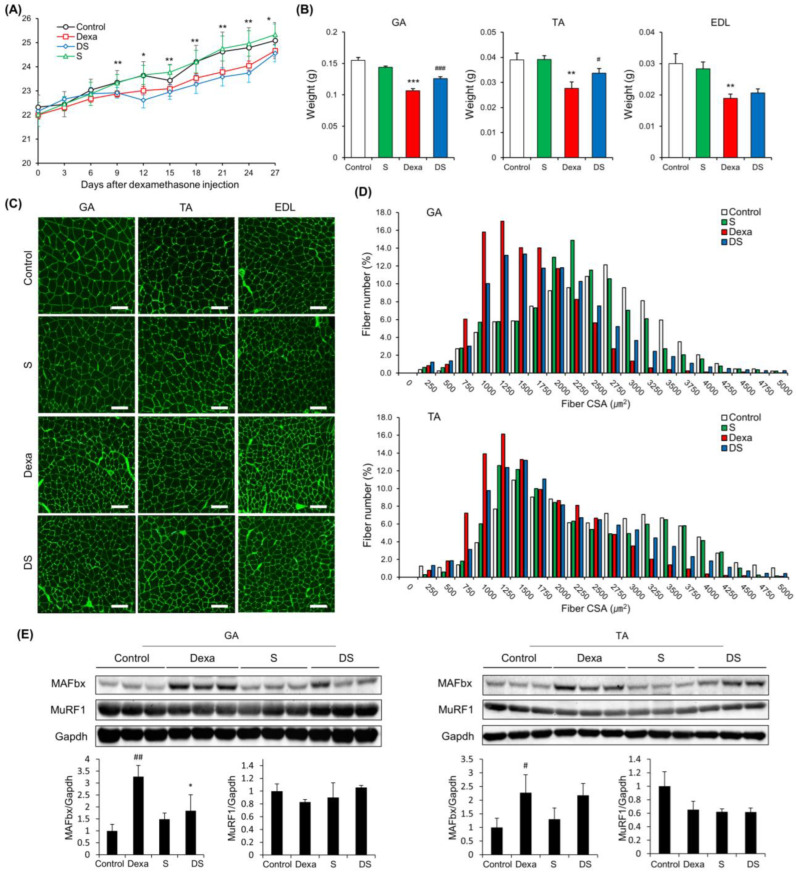
β-sitosterol prevents Dexa-induced muscle atrophy in mice. (**A**) Body weight. * *p* < 0.05 and ** *p* < 0.01 control vs. Dexa. (**B**) Muscle weight of GA, TA, and EDL. ** *p* < 0.01 and *** *p* < 0.001 vs. control. # *p* < 0.05 and ### *p* < 0.001 vs. Dexa. (**C**) Representative immunofluorescent staining of myofiber cross section of GA, TA, and EDL. A microscope with a 10× objective was used to capture the images. The scale bar represents 100 μm. (**D**) Quantification of myofiber size by cross-sectional area (CSA) measurements for GA and TA muscle. Data are shown as mean ± S.E. (*n* = 9 per group). (**E**) Representative images of the western blot analyses for MAFbx and MuRF1 in GA and TA. # *p* < 0.05, ## *p* < 0.01 vs. control; * *p* < 0.05 vs. Dexa. GA—gastrocnemius muscles; TA—tibialis anterior; EDL—extensor digitorum longus; Dexa—Dexamethasone; S—β-sitosterol; DS—dexa + β-sitosterol.

**Figure 2:**
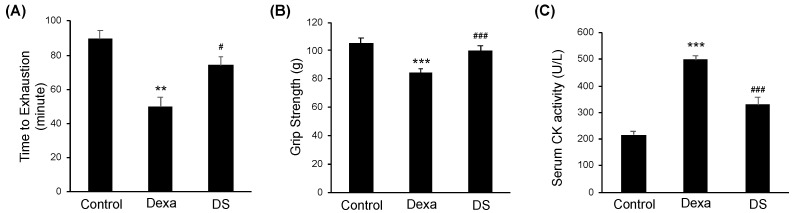
β-sitosterol ameliorates Dexa-induced muscle dysfunction in mice. (**A**) Forced running time. (**B**) Grip strength. (**C**) Serum CK activity. ** *p* < 0.01 and *** *p* < 0.001 compared to the untreated control. # *p* < 0.05, ### *p* < 0.001 compared to the to the Dexa treated control. DS—dexa + β-sitosterol; CK—creatine kinase.

There was a significant difference in body weight between the Control group and the Dexa group after dexamethasone administration (Figure 1A). Next, the backfoot muscles, the gastrocnemius, and tibialis anterior muscles were cut off and weighed. As a result, the weight of these muscles was dramatically reduced in the Dexa group. However, in the DS group, it weighed more compared to the Dexa group. The extensor digitorum longus also showed similar results, but there were no significant differences between the Dexa group and the DS group (Figure 1B). Generally, sarcopenia leads to the loss of muscle mass, which is associated with reduced muscle fiber number and size. Therefore, to assess the histological properties of the morphological change of the muscle fibers, immunofluorescence staining was done on the tibialis anterior muscle, the gastrocnemius muscle, and extensor digitorum longus muscle of the muscle atrophy mouse model. In all three types of muscles, the size of muscle fibers decreased in the Dexa group and recovered in the DS group (Figure 1C). In the quantification of myofiber size of gastrocnemius muscles and tibialis anterior muscles, total muscle fiber atrophy in the Dexa group resulted in a decrease in muscle fiber thickness when compared to the control group. On the other hand, the gastrocnemius muscle of the DS group showed an increase in muscle fiber thickness (Figure 1D). Because all catabolic pathways of skeletal muscle induce up-regulation of MuRF-1 and MAFbx, we investigated the effect of muscle catabolism by evaluating changes in the expression of MuRF-1 and MAFbx [46]. Changes in protein expression of MAFbx and MuRF according to β-sitosterol treatment were confirmed in gastrocnemius muscle and tibialis anterior muscle. MAFbx increased in the Dexa group and decreased in the DS group, but there was no significant difference (Figure 1E). The time to exhaustion of treadmill exercise was dramatically accelerated in the Dexa group but recovered in the DS group in an experiment comparing the time to exhaustion of treadmill exercise conducted with the dexamethasone-induced muscle atrophy mouse model (Figure 2A). Grip strength was also decreased in the Dexa group compared to the control group and recovered in the DS group (Figure 2B). Likewise, serum creatine kinase (CK) activity was increased in the Dexa group and recovered in the DS group (Figure 2C).

### 3.2. In Vitro Verification of Muscle Loss Inhibitory Efficacy of β-Sitosterol in Dexamethasone-Induced Muscle Atrophy in C2C12 Myotube

**Figure 3:**
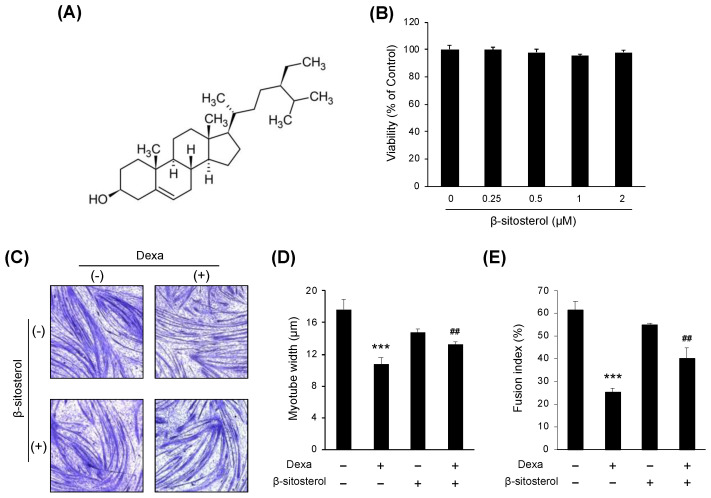
β-sitosterol attenuates Dexa-induced muscle atrophy in C2C12 myotubes. (**A**) Chemical structure of β-sitosterol. (**B**) C2C12 cells were cultured in medium with various concentrations (0.25, 0.5, 1, and 2 mM) of β-sitosterol for 24 h. Cell viability was measured by a CCK-8 assay. The viability of control (untreated cells) was set to 100%. Viability as a percentage of that of control cells is shown. Bars represent the mean ± S.D. (*n* = 4 per treatment). (**C**) May–Grunwald and Giemsa staining. The cells were incubated with 0.5 mM β-sitosterol for 48 h in the presence or absence of 1 μM dexamethasone (Dexa) for 48 h. (**D**) Quantification of myotube widths from May–Grunwald and Giemsa stained images. Data shown are mean ± S.D. (*n* = 100). *** *p* < 0.001 vs. untreated control; ## *p* < 0.01 vs. Dexa. (**E**) Fusion index. The total number of nuclei incorporated in myotubes and the total number of nuclei were scored. Fusion index was calculated as the percentage of total nuclei incorporated in myotubes. *** *p* < 0.001 vs. untreated control, ## *p* < 0.01 vs. Dexa. DS—dexa + β-sitosterol.

**Figure 4:**
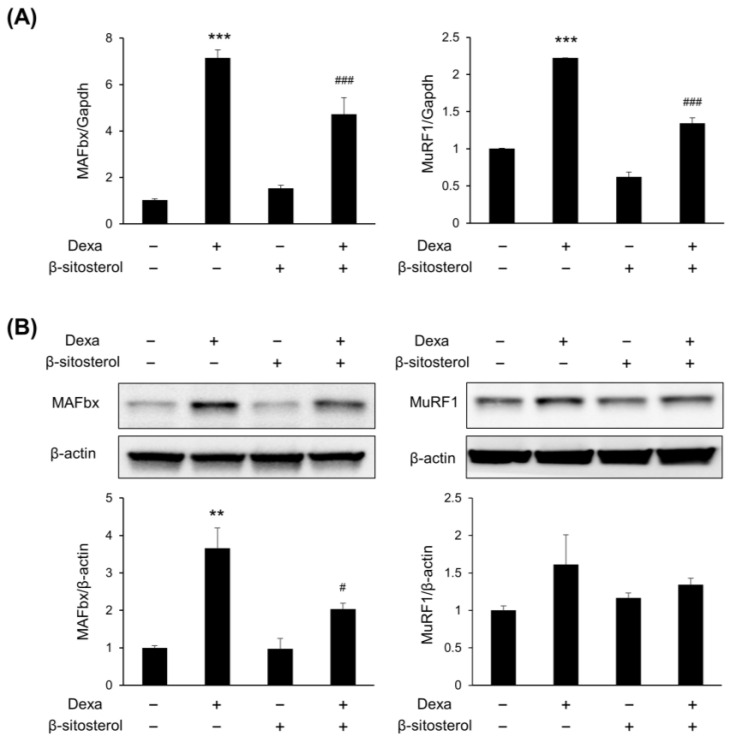
β-sitosterol suppresses the expression of MAFbx and MuRF1. C2C12 myotubes were treated with 0.5 mM β-sitosterol in the presence or absence of 1 μM Dexa for 48 h. (**A**) MAFbx and MuRF1 mRNA levels were analyzed by quantitative PCR. GAPDH was used as a control. Data are shown as mean ± S.D. (*n* = 4 per group). *** *p* < 0.001 vs. control; ### *p* < 0.001 vs. Dexa. (**B**) The expression of MAFbx and MuRF1 protein in C2C12 myotubes was estimated by Western blot analysis. β-actin was used as a control for protein loading. ** *p* < 0.01 vs. control, # *p* < 0.05 vs. Dexa.

β-sitosterol was evaluated in C2C12 myotubes differentiated from C2C12 myoblasts to find small molecules that protect muscles from dexamethasone-induced muscle atrophy (Figure 3A). There was no significant difference in the viability of cells even when sitosterol was added to the C2C12 culture medium with various concentrations (0.25, 0.5, 1, and 2 mM) (Figure 3B). The myotube width was significantly decreased in the dexamethasone treatment group, but it was increased in the dexamethasone + β-sitosterol treatment group (Figure 3C,D). In the fusion index, the total nuclei of myotube were significantly decreased in the Dexa group but recovered in the DS group (Figure 3E). The gene expression of sarcopenia-associated genes of myotube was evaluated. In the dexamethasone treatment group, the expression of MuRF1 and MAFbx was increased. However, in the dexamethasone + β-sitosterol 500 μM treatment group, it was reduced (Figure 4A). The protein expression of MuRF1 and MAFbx was also evaluated. The protein expression of MAFbx showed a significant increase in the dexamethasone treatment group and a significant decrease in the dexamethasone + β-sitosterol group. MuRF1 also showed similar results to MAFbx but did not show a significant difference (Figure 4B).

### 3.3. β-Sitosterol Inhibited FoxO1-Mediated Protein Degradation, Reducing Dexamethasone-Induced Atrophy

**Figure 5:**
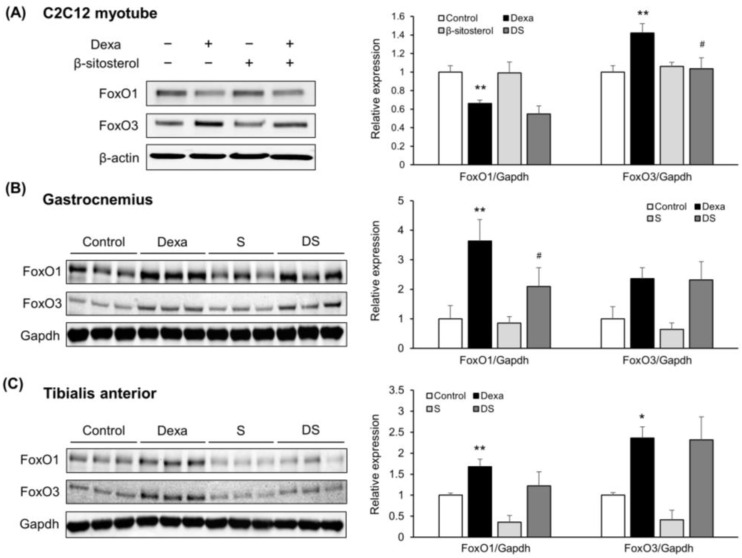
β-sitosterol inhibits Dexa-induced atrophy in C2C12 myotube and mice via FoxO1/3-dependent signaling. (**A**) Representative images of the Western blot analyses for FoxO1 and FoxO3 in C2C12 myotube. Right panel show quantification of the indicated proteins. Data shown are mean ± S.D. (*n* = 3 per group). ** *p* < 0.01 vs. control; # *p* < 0.05 vs. Dexa. (**B**) Representative images of the Western blot analyses for FoxO1 and FoxO3 in gastrocnemius muscles. Right panel show quantification of the indicated proteins. Data shown are mean ± S.D. (*n* = 3 per group). ** *p* < 0.01 vs. control; # *p* < 0.05 vs. Dexa. (**C**) Representative images of the Western blot analyses for FoxO1 and FoxO3 in tibialis anterior muscles. Right panel show quantification of the indicated proteins. Data shown are mean ± S.D. (*n* = 3 per group). ** *p* < 0.01; * *p* < 0.05 vs. control. Dexa—Dexamethasone; S—β-sitosterol; DS—dexa + β-sitosterol.

Next, FoxOs, which is an up-regulator of MuRF1 and MAFbx, expression levels were measured in the C2C12 myotube [47]. First, FoxO1 decreased in the dexamethasone treatment group. In contrast, FoxO3 protein was increased in the dexamethasone treatment group, and the expression level increased when β-sitosterol was administered (Figure 5A). However, in the gastrocnemius and tibialis anterior muscles, FoxO1 protein was increased in the Dexa group compared with the control group and reduced in the DS group. FoxO3 protein was increased in the Dexa group, but there were no changes in the DS group (Figure 5B,C). These results showed that the FoxO3 muscle atrophy-related signal transduction pathway is not thought to be a regulatory mechanism for β-sitosterol’s protective action. Therefore, β-sitosterol prevents dexamethasone-induced muscle atrophy, and β-sitosterol is expected to play a key role in limiting the ubiquitin-proteasome pathway triggered by reducing FoxO1.

## 4. Discussion

β-sitosterol is a bioactive phytosterol found in plant cell membranes and has a molecular composition comparable to cholesterol found in human cells [48]. β-sitosterol and cholesterol have similar structures, but β-sitosterol has an extra ethyl group at C-24. Rhamnol, cinchol, cupreol, sitosterin, quebrachol, (3β)-stigmast-5-en-3-ol, 22:23-dihydro stigmasterol, and α-dihydrofucosterol are some of the other names for β-sitosterol. It comes in three different forms: anhydrous, hemihydrate, and monohydrate, depending on the number of water molecules added [16]. β-sitosterol is known to regulate factors related to apoptosis, immunological response, anticancer defenses, and inflammation [49,50]. Particularly, antioxidants, both enzymatic and non-enzymatic, are increased by β-sitosterol, making it a potent antidiabetic, hypolipidemic, neuroprotective, and chemopreventive agent [51].

Several studies demonstrated that the concentration of plasma β-sitosterol was shown to be considerably lower in type 2 diabetes patients, suggesting that β-sitosterol may play a role in decreasing blood glucose levels [52]. The study of Pandey et al. reported that β-sitosterol-D-Glucopyranoside (BSD) extracted from Cupressus sempervirens stimulates estrogenic analog effects and glucose uptake, affecting skeletal muscle cells. They found that BSD-induced GLUT4 translocation stimulates skeletal muscle cells by increasing glucose uptake through the PI-3K/AKT mechanism [53].

In skeletal muscle, β-sitosterol has been studied to improve mitochondrial ATP production’s responsiveness to increased energy demand. Hoi Shan Wong (2015) et al. reported that β-sitosterol extracted from Citanches Herba fraction (HCF1) induces redox-sensitivity induction of mitochondrial segregation in C2C12 myotubes and activation of AMPK/PGC-1. They did not report the results of animal experiments on the inhibition of muscle loss but demonstrated the anti-obesity effect of body energy expenditure in skeletal muscle in high fat-dieted, obese-induced mice [54]. After that, Hoi Shan Wong et al. and others reported through further study that β-sitosterol can increase the reaction of mitochondrial membrane to fluidization and energy demand in the mitochondrial electron transfer system, thereby suppressing mitochondrial-related muscle function degradation by not affecting C2C12 muscle separation induction [55]. The fact that there are many investigations of antioxidant-related effects in prior studies is also thought to disprove the results of the study. On the contrary, Hwang et al. investigated the regulation of fat and glucose metabolism by the AMP-activated protein kinase of β-sitosterol. Their experiments using L6 myotube cells demonstrated that β-sitosterol enhances TG inhibition and glucose uptake through the AMPK mechanism. Their findings are contrary to the results of our AMPK inhibitory effect, and it seems necessary to analyze the differences in muscle cell lines and research settings used in cell experiments [52].

**Figure 6:**
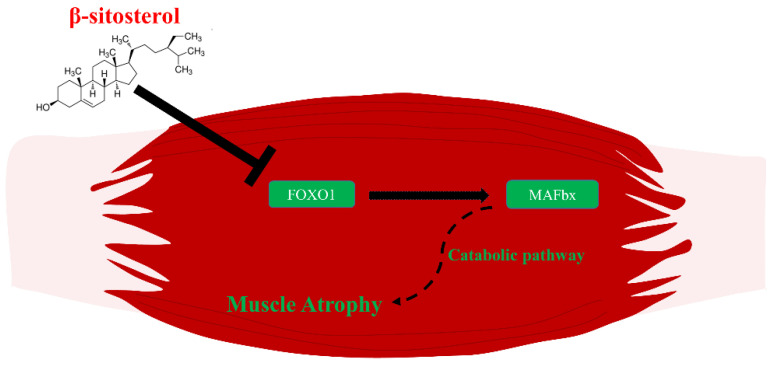
The overall β-sitosterol mechanisms related to muscle atrophy. β-sitosterol down-regulates the transcriptional factor, FoxO1. Down-regulated FoxO1 becomes unable to affect the expression of MAFbx. Consequently, inhibited MAFbx cannot induce muscle atrophy via the catabolic pathway.

The present study found that β-sitosterol inhibited muscle atrophy in dexamethasone-treated muscle atrophy cells and animal models, a model of catabolism-induced muscle atrophy, which is one of the major mechanisms of aging sarcopenia. These results suggested that blocking the ubiquitin-proteasome pathway induced by suppressing FoxO1, which is an up-regulator of MAFbx, plays an important role (Figure 6). In addition, β-sitosterol was reported in relation to foxo1 regulating the catabolic pathway by promoting several genes, including MAFbx expression [33,56,57]. Therefore, β-sitosterol might inhibit the catabolic pathway by downregulating FoxO1/MAFbx, thereby preventing protein catabolism, which is key to aging muscle loss. However, in vitro experiment of C2C12 myotube showed controversial results that FoxO1 was decreased in the dexamethasone treatment group. Therefore, further studies related to the in vitro experiment on FoxO1 protein expression is needed.

In aging sarcopenia, it may be difficult to achieve desired muscle strength improvement and muscle mass increase even with appropriate exercise due to inflammation and degeneration of neuromuscular junctions. Nevertheless, the mechanism of the study of the mitochondrial electron transport system conducted by Wong et al. and the mechanism of inhibition of muscle degradation by blocking the ubiquitin-proteasome pathway demonstrated in our study proves the important possibility of using β-sitosterol for the inhibition of aging sarcopenia [54,55].

## 5. Conclusions

In the present study, we found that β-sitosterol has anticatabolic effects in skeletal muscles by regulating the FoxO1/MAFbx pathway, which causes muscle loss. This result was confirmed in dexamethasone-treated muscle atrophy C2C12 myotube and mouse models. Therefore, β-sitosterol would be a potential treatment agent for aging sarcopenia.

## Data Availability

The data presented in this study are available on request from the corresponding author.
